# Clinical evaluation of a spot-check ECG for the diagnosis of atrial fibrillation

**DOI:** 10.1016/j.hroo.2026.01.006

**Published:** 2026-01-13

**Authors:** Cameron Lambert, Jay Ganji, Ahmed Alghazi, Wilson W. Good, Elyar Ghafoori, Daniel J. Cantillon, Paul Drury

**Affiliations:** 1 Cone Health, Greensboro, North Carolina; 2 Masimo Corporation, Irvine, California; 3 PCA Cardiology Associates, Laguna Hills, California

**Keywords:** Atrial fibrillation, Early detection of disease, Wearable devices, Ambulatory electrocardiographic monitoring, Cardiac arrhythmias

## Abstract

**Background:**

Opportunistic screening of atrial fibrillation (AF) using wearable spot-check electrocardiogram (ECG) devices has emerged for early detection, yet remains limited by concerns for accuracy and false positives.

**Objective:**

This study aimed to assess the Masimo W1 wrist-wearable ECG spot-check device in detecting AF and normal sinus rhythm (NSR) while rendering diagnostic ECG waveform quality to support physician overread.

**Methods:**

Participants with and without a previous AF diagnosis were prospectively enrolled across 4 US sites to compare the AF and NSR detection performance between the Masimo W1 ECG and a 12-lead ECG reference, plus concordance of clinically relevant ECG waveform features assessed by blinded reviewers.

**Results:**

Among 356 participants (mean age 56 years; 60% female), 169 (47.5%) were in NSR and 165 (46.3%) in AF, whereas 22 were excluded as noise outputs (1.7%) or other rhythms (4.5%). Sensitivity and specificity for automated AF detection were 99.3% (95% confidence interval [CI] 96.3–100) and 100% (95% CI 97.8–100), respectively. The inconclusive rate was 5%. AF and NSR detection probabilities were 89.7% and 99.4%, respectively. Qualitative comparison demonstrated 99% (95% CI 98–100.0) agreement with the reference ECG across reviewers and records. Quantitative analysis of ECG waveform features showed a high correlation between the Masimo W1 ECG and lead I of the 12-lead ECG for diagnosing AF.

**Conclusion:**

The Masimo W1 ECG demonstrated highly accurate automated AF detection, few noise classifications, and exceptional NSR detection to mitigate concerns over false positives. Qualitative preservation of ECG waveform features supports the requisite physician overread required by practice guidelines.

## Graphical abstract

**Figure:**
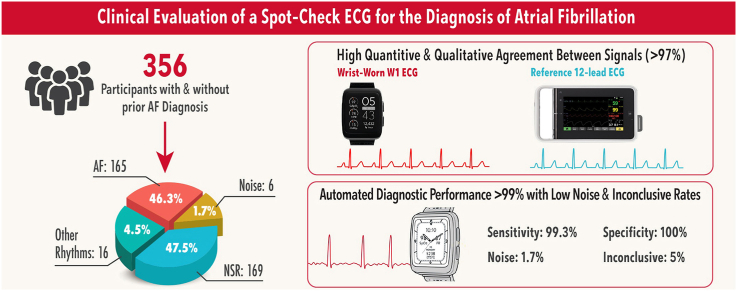

**Key Findings**

▪ Opportunistic screening using wearable spot-check electrocardiogram (ECG) devices has emerged as a means of early detection of atrial fibrillation (AF), yet remains limited by concerns for accuracy and false positives.
▪ Current practice guidelines stipulate visual inspection of the ECG waveform by a qualified clinician to confirm the diagnosis prior to initiating pharmacologic stroke.
▪ The W1 ECG demonstrates highly accurate automated AF detection, few noise classifications, and exceptional normal sinus rhythm detection to mitigate concerns over false positives.
▪ Qualitative preservation of ECG waveform features by blinded manual adjudication supports the requisite physician overread required by current clinical practice guidelines.

## Introduction

Atrial fibrillation (AF) is estimated to affect up to 12 million people in the United States by 2030 and is associated with an increased risk of stroke, myocardial infarction, heart failure, dementia, and mortality.^1^ Recent European Society of Cardiology practice guidelines advocate opportunistic screening for early detection, risk factor modification, and stroke prevention strategies.^2^ Stroke can be the first clinical manifestation of AF in many asymptomatic or minimally symptomatic individuals. Moreover, up to 30% of patients with embolic stroke of uncertain origin demonstrate AF during extended monitoring using implantable loop recorders.^2^^,^^3^ Recently updated American College of Cardiology/American Heart Association/American College of Clinical Pharmacy/Heart Rhythm Society practice guidelines identify wearable and consumer-accessible electrocardiogram (ECG) spot-check devices as a potential tool for earlier AF detection, although the authors raise concerns regarding automated detection accuracy, including false positives that lead to unnecessary clinical evaluation and patient worry.^4^ These guidelines stipulate visual inspection of the ECG waveform by a qualified clinician to confirm the diagnosis before initiating pharmacologic stroke prophylaxis. However, a recent study involving Food and Drug Administration–cleared and Conformité Européenne–marked devices demonstrated a limited ability of clinicians to accurately confirm AF using tracings obtained from single-lead ECG spot-check devices, with only 72% sensitivity and 92% specificity for cardiologists and substantially lower results for internists and medical students.^5^ Thus, it is very important to render a high-quality ECG waveform of diagnostic quality for over-reading physicians.^6^

The emergence of consumer-accessible spot-check ECG devices, which include portable ECG monitors and wrist-wearable and smartphone-connected ECG tools, has irreversibly altered the technological landscape of cardiac monitoring by increasing accessibility to patients.^7^ Unlike traditional ECG devices that require a clinical setting and sophisticated equipment, consumer-based ECG devices allow individuals to perform self-directed heart rhythm assessments as they see fit. In addition, wearable spot-check devices can be a more cost-effective solution for AF screening than no screening or the use of traditional screening modalities.^8^ The goal of consumer-accessible devices is to facilitate early AF detection, appropriate medical consultations, and timely interventions. Despite such promise, the clinical efficacy and accuracy of consumer-based ECG devices in diagnosing AF and the fidelity of the waveforms provided by these devices can vary widely. A recent comparative study among consumer-accessible ECG devices demonstrated sensitivity and specificity for diagnosing AF as low as 58% and 69%, respectively, and inconclusive rates as high as 26%.^9^

The present study will evaluate whether the spot-check ECG feature of the Masimo W1 wrist-wearable device provides accurate automated detection of AF and normal sinus rhythm (NSR) while rendering diagnostic ECG waveform quality for manual adjudication similar to lead I of a 12-lead reference ECG.

## Methods

### Study design and participant selection

In this single-arm, multicenter prospective study (NCT06071754), participants were enrolled across 4 clinical study sites (site 1, Masimo, Irvine, CA; site 2, Piedmont Cardiovascular, Greensboro, NC; site 3, PCA Cardiology Associates, Laguna Hills, CA; site 4, Cone Health, Greensboro, NC). Participants included (1) individuals without a previous arrhythmia diagnosis presenting in NSR and (2) individuals with a history of paroxysmal or persistent AF presenting in AF. All participants were 22 years or older. The exclusion criteria were individuals with implantable defibrillators or cardiac pacing devices, a previous diagnosis of non-AF cardiac arrhythmia, open wounds or skin conditions that would preclude proper device placement, known allergic reactions to adhesive tapes or ECG gel, or inability to physically wear a wristwatch.

The study was reviewed and approved by Western-Copernicus Group Institutional Review Board (Puyallup, Washington) and Cone Health Institutional Review Board (Greensboro, North Carolina). The study was performed according to the principles of the Declaration of Helsinki, and all participants provided a written informed consent.

### Masimo W1 ECG

This study used the Masimo W1, a wrist-wearable health tracking device. The Masimo W1 provides a 30-second spot-check ECG, similar to a lead I ECG, that can support automated AF detection and ECG-derived heart rate (HR). In addition, Masimo W1 offers continuous photoplethysmography (PPG), enabling continuous monitoring of pulse rate, blood oxygen saturation, and other parameters. ECG measurements are enabled by users selecting the ECG option from the menu and touching the Masimo W1’s bezel by forming a C with the index finger and thumb from the opposite hand. The user is then instructed to keep their fingers in the same position until the 30-second ECG is completed. The electrodes located on the back of the device, along with the bezel electrode, function similarly to the limb lead I electrodes between the left and right arms. Once complete, Masimo W1 displays the automated rhythm classification and HR. In addition, a PDF is generated that provides the 30-second ECG, along with the participant’s record information, automated detection results, and HR. This PDF can be shared with the participant’s health care provider for further evaluation.

### Study procedure

Each participant underwent a routine 12-lead ECG and a contemporaneous ECG spot-check measurement from the Masimo W1 device. The principal investigator (PI) at each site reviewed the 12-lead ECG to establish the reference ECG rhythm classification (ground truth). The concurrent 10-second standard 12-lead ECG and the 30-second ECG PDFs from the Masimo W1 were digitally stored for further analysis.

### Automated detection analysis

The automated detection outputs from the Masimo W1 ECG were categorized as follows:

1. NSR: the Masimo W1 ECG output was NSR.
2. AF: the Masimo W1 ECG output was AF.
3. Unclassified: the Masimo W1 ECG output was inconclusive, or the HR was outside the detection range (high or low HR for NSR [50–100 beats per minute {bpm}] or AF [50–150 bpm]).
4. Noise: the Masimo W1 ECG output was noise (when waveform quality is insufficient).

To evaluate the AF detection of the Masimo W1 ECG, each record was assigned to 1 of 4 reference classifications. These categories were based on the clinical interpretation of the 12-lead ECG by the PI at each site. The cardiac rhythm classifications of the reference ECG were defined as follows:

1. NSR: the PI adjudicated the rhythm as NSR with an HR ranging between 50 and 100 bpm.
2. AF: the PI adjudicated the rhythm as AF with an HR ranging between 50 and 150 bpm.
3. Other: the PI did not adjudicate the rhythm as either AF or NSR, or the HR was outside the range detectable by the Masimo W1.
4. Uninterpretable: the quality of the 12-lead ECG was insufficient for an accurate diagnosis.

### Automated detection performance evaluation

The established references based on the adjudicated 12-lead ECG results were compared with Masimo W1 ECG’s automated rhythm classification. The sensitivity and specificity of the Masimo W1 ECG’s automated classification of AF were calculated and compared with the PI’s diagnosis of the 12-lead ECG. Furthermore, the inconclusive rate was calculated as the rate at which Masimo W1 classified ECGs as “unclassified” whereas the adjudicated rhythm from the 12-lead ECG was either NSR or AF. Separately, the rate at which Masimo W1 could not classify owing to the presence of noise was reported.

### Waveform qualitative analysis

The diagnostic capability of the Masimo W1 ECG waveform was evaluated by comparing the rhythm diagnosis made by 3 independent physicians, who were blinded to the adjudicated rhythm from the standard 12-lead ECG determined by the PI at each site. For the qualitative assessment, the percentage of agreement for each observer and across observers was calculated and reported. Similarly, the mean error for each quantitative measurement was reported per observer and across observers. In addition, each of the 3 physicians independently assessed the presence or absence of the P wave on both the Masimo W1 ECG and the 12-lead ECG. These 2 independent assessments were conducted to qualitatively compare the diagnostic effectiveness of the Masimo W1 ECG with the standard 12-lead ECG in detecting AF.

### Waveform reproducibility quantitative analysis

**Figure 1:**
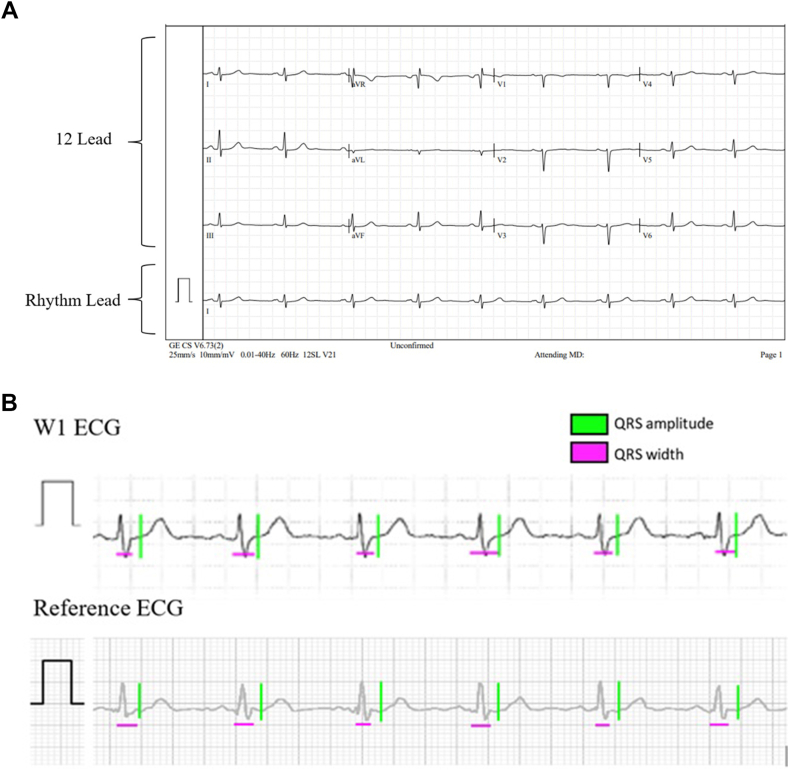
**A:** Reference 12-lead ECG simultaneously acquired to Masimo W1 ECG. The rhythm lead was selected to lead I. **B:** An example of quantitative analysis on the Masimo W1 ECG (top) and lead I (bottom), where QRS amplitude and width were annotated on the first 6 native beats. ECG = electrocardiogram.

The time intervals and amplitudes were quantitatively measured by 3 independent experts on the first native beats on both Masimo W1 ECG and lead I of the 12-lead ECG to evaluate similarities between Masimo W1 ECG and lead I of the 12-lead ECG with respect to clinically relevant features, including QRS amplitude and QRS width (Figure 1).

### Sample size calculation

The sample size was determined to meet simultaneous requirements for both sensitivity and specificity analysis. The PASS 2019 software package (NCSS, Kaysville, UT) was used to calculate the appropriate sample size using the confidence intervals (CIs) for a 1-sample sensitivity and specificity module. The exact (Clopper-Pearson) formula was used to provide a 97.5%, 1-sided, CI with the lower bound of the CI to be greater than 0.90 with a power of >0.80. Given that the participants were recruited into 1 of 2 groups (with or without a previous AF diagnosis), the expected prevalence of AF in this sample population was set to 50%. To achieve a power of >0.80, a minimum sample size of 118 subjects in each group (AF and NSR) meets the CI width, power, and minimum CI lower bound requirements. 20% was added to this sample size to account for inconclusive, noise, and other rhythms in the subjects being sampled. The total required sample size was assessed to be a minimum of 284 total (118∗2∗1.2) for the analysis.

### Statistical analysis

The automated detection performance of the Masimo W1 ECG was evaluated by determining the percentage of successful automated rhythm classifications compared with the ground truth using the adjudicated 12-lead ECG, and the sensitivity and specificity for detecting AF were calculated. Additional calculations included the rates at which the automated rhythm classifications were “unclassified” or “noise” when the reference was deemed to be NSR or AF, as well as the AF detection probability and NSR detection probability of the Masimo W1 ECG.

The Masimo W1 ECG waveform was qualitatively assessed by determining the percentage agreement for rhythm classification, manually adjudicated by blinded physicians, in comparison with the ground truth using the adjudicated 12-lead ECG results. The percentage of agreement for each observer and across observers was calculated and reported. In addition, the percentage agreement for the presence or absence of the P wave between the Masimo W1 and the 12-lead ECG was determined. For the quantitative assessment of the Masimo W1 ECG waveform, the mean differences in QRS width and QRS amplitude were calculated, as measured by 3 independent experts on the first native beats of the Masimo W1 ECG and lead I of the 12-lead ECG.

## Results

### Participant demographics

A total of 356 participants (mean age 56 years; 60% female) were enrolled across 4 sites (Masimo, 176; Piedmont Cardiovascular, 46; PCA Cardiology Associates, 80; Cone Health, 46), including 176 individuals with no history of AF and 180 individuals with diagnosed AF (Table 1). All 176 participants without a history of AF presented in NSR during the study, although 1 participant within this group presented with frequent premature ventricular contractions. Among the 180 participants with diagnosed AF, 178 were in AF at the time of the study. 1 participant in this group was in NSR, whereas another presented with atrial flutter during the study period.

**Table 1:** Demographic and baseline clinical characteristics of the study cohort (n = 356 participants)

Characteristics	Participants with a previous AF diagnosis	Participants with no history of AF	All participants
N	180	176	356
Sex (M/F)	124 (68.9)/56 (31.1)	98 (55.7%)/78 (44.3)	222 (62.4)/134 (37.6)
Age	74 ± 9	66 ± 10	56 ± 21
Race			
Asian	5 (2.8)	51 (29)	56 (15.7)
Black	9 (5)	16 (9.1)	25 (7)
Native Hawaiian Islander	0 (0)	2 (1.1)	2 (0.6)
White	130 (72.2)	72 (40.9)	202 (56.7)
Other	0 (0)	33 (18.8)	33 (9.3)
Not provided	36 (20)	2 (1.1)	38 (10.7)
Ethnicity			
Hispanic	3 (1.7)	51 (29)	54 (15.2)
Non-Hispanic	132 (73.3)	120 (68.2)	252 (70.8)
Not provided	45 (25)	5 (2.8)	50 (14)
Weight (lb)	205 ± 54	174 ± 38	190 ± 50
Height (in)	69 ± 4	67 ± 4	68 ± 58
BMI	30 ± 7	27 ± 5	29 ± 6

Values are given as n (%) or mean ± standard deviation.

AF = atrial fibrillation; BMI = body mass index; F = female; M = male.

### Automated detection performance evaluation

Of the 356 participants, 169 (47.5%) were determined to be in NSR with an HR between 50 and 100 bpm, whereas 165 participants (46.3%) were found to be in AF with an HR between 50 and 150 bpm. The remaining 22 participants were excluded for having an HR value falling outside the automated detection range (n = 14; 3.9%), a “noise” output by the Masimo W1 (n = 6; 1.7%), or an adjudicated rhythm from the 12-lead ECG that was neither AF nor NSR (n = 2; 0.6%). There were no participants for whom the reference ECG was unreadable.

Of the 169 participants with confirmed NSR diagnosis, the Masimo W1 ECG successfully detected NSR in 168 participants (99.4%), whereas 1 participant (0.6%) was labeled as “unclassified” despite having an HR within the detection range (Table 2A). Among the 165 participants with a confirmed AF diagnosis, the Masimo W1 ECG successfully detected AF in 148 participants (89.7%), whereas 16 (9.7%) were labeled as “unclassified” despite an HR falling within the detection range. There was only 1 participant (0.6%) with reference ECG classification of AF that was misclassified as NSR.

**Table 2:** Performance results of the Masimo W1 automated ECG spot-check detection algorithm according to output classification using a 4 by 4 matrix (A) and results (B)

A) Test reference	Normal sinus rhythm	Atrial fibrillation	Unclassified
Normal sinus rhythm	168	0	1
Atrial fibrillation	1	148	16

B) Performance metrics	Results

Sensitivity	99.3% (95% CI 96.3–100.0)
Specificity	100.0% (95% CI 97.8–100.0)
Inconclusive rate	5.0%
Noise rate	1.7%

CI = confidence interval; ECG = electrocardiogram.

Consequently, the sensitivity for detecting AF using Masimo W1 ECG was 99.3% (95% CI 96.2–100.0), with a specificity of 100% (95% CI 97.8–100.0) (Table 2B). The rate at which the Masimo W1 did not classify the rhythm when the reference ECG was deemed to be NSR or AF, the inconclusive rate, was determined to be 5%. In addition, the rate at which the Masimo W1 ECG failed to provide any classification owing to noise was calculated at 1.7%. The AF detection probability was 89.7%, and the NSR detection probability was 99.4%. Patients with an inconclusive output were older than the remaining cohort (mean age 73.3 vs 55.6; *P* < .005).

### Waveform reproducibility qualitative analysis

The percentage agreement for Masimo W1 ECG rhythm classification, manually adjudicated by blinded physicians, in comparison with the ground truth using the adjudicated 12-lead ECG results, was 99% (95% CI: [98%, 99%]) (Table 3). The percentage agreement for the presence or absence of the P wave, as manually assessed by 3 physicians between the Masimo W1 ECG and the 12-lead ECG, was calculated to be 97% (95% CI 96–98%) (Table 4).

**Table 3:** Percentage agreement for Masimo W1 ECG rhythm classification, manually adjudicated by blinded physicians, in comparison with the ground truth using the adjudicated 12-lead ECG results

Observer	Percent agreement (W1 ECG with adjudicated 12-lead ECG results)
1	100% (95% CI 98–100)
2	98% (95% CI 95–99)
3	98% (95% CI 96–100)
Overall	99% (95% CI 98–99)

CI = confidence interval; ECG = electrocardiogram.

**Table 4:** Percentage agreement per observer for P-wave visibility, manually assessed by blinded physicians, between Masimo W1 ECG and 12-lead ECG

Observer	Percent agreement (W1 ECG with 12-lead ECG)
1	99% (95% C 97–100)
2	97% (95% CI 94–98)
3	97% (95% CI 94–99)
Overall	97% (95% CI 96–98)

CI = confidence interval; ECG = electrocardiogram.

### Waveform reproducibility quantitative analysis

The mean difference in QRS width was found to be 0.1 mm, and the QRS amplitude was found to have a mean difference of 2.6 mm. The QRS waveform of the Masimo W1 ECG, on average, exhibited a 2.6 mm greater amplitude than lead I of the reference 12-lead ECG, suggesting a higher signal-to-noise ratio than the reference, which is considered both expected from a technical perspective and clinically desirable. This difference met the 1-sided (more than −2mm) acceptance criteria. The difference was likely attributed to variations in electrode placement between the reference (torso) and Masimo W1 (wrist), resulting in a larger axis for the Masimo W1. The quantitative similarities between the Masimo W1 ECG and reference ECG waveforms were consistent across operators and were unaffected by the underlying rhythm.

## Discussion

This manuscript reports the results of the single-arm, multicenter premarket study designed to prospectively evaluate the automated AF detection of the Masimo W1 ECG device and establish equivalence between the Masimo W1 and lead I of a standard 12-lead ECG. The principal finding is that the Masimo W1 spot-check ECG demonstrated highly accurate automated single ECG lead detection with 99.3% sensitivity, 100% specificity, an inconclusive rate of 5%, and a noise rate of 1.7% overall. The AF detection probability was 89.7%, and the NSR detection probability was 99.4%. A comparative analysis of these results with similar premarket approval data reported by other wrist-wearable devices with ECG spot-check detection revealed that the overall sensitivity, specificity, and AF detection probability fall within the performance range of similar devices from other manufacturers.^10^, ^11^, ^12^ However, the Masimo W1 ECG noise output of 1.7% is lower than the 6.9%–16.8% noise output range reported in these premarket studies. The inconclusive rate of 5% also falls below the range of 12.2%–19.7% reported by manufacturers of similar wrist-wearable devices.^10^, ^11^, ^12^ The higher mean age among patients with an inconclusive output merits further investigation and should be evaluated as a potentially generalizable concern across wrist-wearable devices. Minimizing noise and inconclusive outputs while preserving diagnostic accuracy is clinically important when considering the potential role of wrist-wearable spot-check ECG devices as an opportunistic screening tool envisioned in practice guidelines.^13^ Moreover, achieving an NSR detection probability as close as possible to 100% may translate into fewer false positive detections, causing patients to needlessly worry or generate unnecessary consultations or communications with their clinician. Previous clinical studies of Masimo’s optical sensors confirmed that the use of signal processing can reduce false alarms for blood oxygen saturation while accurately reading through motion, oxygen desaturation, and low perfusion across a plurality of skin tones.^14^, ^15^, ^16^ Future studies are needed to determine whether comparable results can be achieved using the applied ECG signal processing techniques on the Masimo W1 device. Nonetheless, design prioritization of mitigating artifacts through filtration of corrupting noise extends the standards of class II medical devices to consumer-accessible products. This design doctrine is consistent with the emphasis placed by the contemporary American College of Cardiology/American Heart Association/American College of Clinical Pharmacy/Heart Rhythm Society guidelines on manual verification of the ECG waveform by a qualified clinician,^4^ given that the reviewing clinician can expect ECG waveform quality similar to what would be encountered in a hospital facility. However, premarket approval studies do not always reflect real-world performance. The comparative analysis published by Mannhart et al^9^ demonstrated that future rigorous real-world testing of the Masimo W1 ECG is needed, with the potential for iterative product enhancements and improvement in the algorithm’s automated detection performance. Clinical outcomes studies are needed to confirm that the W1 can be used effectively within its classification as a class II medical device with connectivity to a telehealth platform, as has been demonstrated with other dual-purpose over-the-counter accessible and prescription devices, such as a spot-check pulse oximeter in reducing 30-day readmissions after joint arthroplasty from 12% to zero.^17^ For the Masimo W1 ECG spot-check feature, the potential prescribed use applications include patients with congestive heart failure, valvular heart disease, or known AF. However, future studies are required to link clinical outcomes within each specified use case.

### Study limitations

Despite advancements in mitigating both noise detections and false positives, the challenge of detecting non-AF pathologic arrhythmias remains. Many non-AF supraventricular tachycardia rhythms feature regularized HRs including atrial flutter. Multilead ECG analysis can facilitate diagnosis in such cases. These limitations apply to all single-lead ECG devices on the market. Recently, some consumer-accessible multilead ECG detection solutions with the potential to classify non-AF supraventricular tachycardia rhythms have emerged with potentially promising results.^18^ However, the optimal balance between ease and accessibility of single-lead spot-check devices as an opportunistic AF screening tool with costlier and more complex multilead alternatives remains unknown. An additional limitation of the present analysis is the lack of enrolled patients with left bundle branch block, although this was not an exclusion criterion in the study.

Future directions include further investigation into whether continuous PPG detection of irregular heart rhythm can uniquely facilitate the Masimo W1 to trigger patients to record a spot-check ECG measurement. Masimo W1 is the only Food and Drug Administration–cleared wrist-wearable device with continuous PPG detection capabilities. Given the often asymptomatic (subclinical) and paroxysmal nature of AF, the combination of these 2 detection capabilities in a single wrist-wearable device may better allow clinicians and patients to assess the cardiac rhythm during periods of sleep, distracting daytime activities, and perhaps quantify overall AF burdens more accurately in a future state. All of the above will require further development efforts, premarket validation testing, and real-world clinical outcomes studies.

## Conclusion

The Masimo W1 spot-check ECG demonstrated highly accurate automated AF detection with few noise classifications and exceptional NSR detection probability to mitigate concerns regarding false positives. Qualitative preservation of ECG waveform features by blinded manual adjudication supports the requisite physician overread required by current clinical practice guidelines.
